# Elucidating the Population Dynamics of Japanese Knotweed Using Integral Projection Models

**DOI:** 10.1371/journal.pone.0075181

**Published:** 2013-09-20

**Authors:** Joseph T. Dauer, Eelke Jongejans

**Affiliations:** 1 School of Natural Resources, University of Nebraska, Lincoln, Lincoln, Nebraska, United States of America; 2 Department of Animal Ecology and Ecophysiology, Radboud University, Nijmegen, The Netherlands; University of Saskatchewan, Canada

## Abstract

Plant demographic studies coupled with population modeling are crucial components of invasive plant management because they inform managers when in a plant’s life cycle it is most susceptible to control efforts. Providing land managers with appropriate data can be especially challenging when there is limited data on potentially important transitions that occur belowground. For 2 years, we monitored 4 clonal Japanese knotweed ( *Polygonum* *cuspidatum* ) infestations for emergence, survival, shoot height until leaf senescence, dry shoot biomass after senescence, and rhizome connections for 424 shoots. We developed an integral projection model using both final autumn shoot height and shoot biomass as predictors of survival between years, growth from year to year, and number of rhizomes produced by a shoot (fecundity). Numbers of new shoots within an infestation (population growth rate λ) were projected to increase 13-233% in a year, with the greatest increase at the most frequently disturbed site. Elasticity analysis revealed population growth at 3 of the 4 sites was primarily due to ramet survival between years and to year-to-year growth in shoot height and shoot biomass. Population growth at the fourth site, the most disturbed, was due to the large production of new rhizomes and associated shoots. In contrast to previous studies, our excavation revealed that most of the shoots were not interconnected, suggesting rhizome production may be limited by the size or age of the plants, resource availability, disturbance frequency, or other factors. Future integration of plant population models with more data on belowground growth structures will clarify the critical stages in Japanese knotweed life cycle and support land managers in their management decisions.

## Introduction

The impact of Japanese knotweed ( *Fallopia* *japonica* *,* *Polygonum* *cuspidatum* *,* *Reynoutria* *japonica* ) has been recognized throughout Europe and North America [1,2]. Riparian and disturbed areas are especially vulnerable to invasion by Japanese knotweed and other weedy species because of an influx of nutrients, plentiful light, and frequent deposition of new propagules [1,3,4]. In addition, Japanese knotweed can establish on a wide variety of soil types [2] and can quickly develop monospecific stands that reduce plant diversity within the infestation [1,5].

The rapid and devastating effects of Japanese knotweed has triggered extensive management efforts with highly variable results. Cutting and digging generally are not advisable because Japanese knotweed can regrow from stem and rhizome fragments as small as 0.7 g [6]. Systemic herbicides show the greatest promise for affecting Japanese knotweed individuals (see Barney et al. 2006 for chemical control review) and rely on carbon movement in the plant to be translocated to the sites of action in new growing points within the plant. One nonchemical management option is *Aphalaraintadori* , a sap-sucking psyllid that prefers Japanese knotweed. The psyllid has been released in the UK and is being investigated for release in the US [7,8]. Like other plant biological control studies [9,10], the success of *A* *. intadori* may hinge on its ability to target the vulnerabilities of the Japanese knotweed life cycle.

Currently there are few models that couple population growth and demography of Japanese knotweed and none of them examine the ramet demography across multiple sites. Suzuki [11] monitored Japanese knotweed patches over time, using shoot counts, to parameterize a reaction diffusion model of spread. Adachi et al. [12] expanded Suzuki’s work to include rhizome growth rates and rhizome branching angles to explain central die-back observed in native, mature Japanese knotweed populations. Smith et al. [13] used demographic data from a single site to parameterize a 3-D growth model of Japanese knotweed in the United Kingdom, comparing to Adachi et al. [12] for differences between the native the invasive varieties. We relied on extensive demographic data [2,6,14] to inform our data collection to parameterize a semi-mechanistic population growth model at multiple sites.

Matrix population models have been constructed for other clonal species, showing in many cases that clonal propagation contributes significantly to population growth [15,16]. The way clonality is incorporated in these models depends on the type of clonal growth and the available data. In tussock-forming plant species, for instance, tussock diameter is often used as the state variable, rendering the process of clonality similar to growth in non-clonal species. For species that also form clonal offspring further away from the existing shoots, e.g., through rhizomes as in Japanese knotweed, the ramets of shoots are often the unit that is modeled (rather than the number of genets in a population). How clonality is included depends most on whether parent-offspring relationships are known between new clonal offspring and last year’s shoots. In slow-growing species like the Mayapple (*Podophyllum peltatum*), where rhizomes are easily visible and long-lived, offspring size can be modeled as a function of parent size based on observational data from demographic plots [17]. When clonal parentage cannot be determined without disturbing the plants too much, clonality rates can simply be estimated by dividing the number of new offspring by the number of ramets in the previous year [18]. However, since clonal offspring production is often a function of parent size and mortality, it is worthwhile to investigate these parent-offspring relationships by excavation. For the clonal thistle *Cirsium* *dissectum* , for instance, belowground investigations outside demographic plots showed that rhizome production was higher in rosettes that flowered and died than in non-flowering rosettes [15].

Understanding clonality and properly incorporating it in population models of the highly clonal Japanese knotweed is difficult but crucial when we want to use such models for studying the population-level effects of local site and treatment effects on vital rates like survival, clonal propagation and sexual reproduction. If these vital rates are size-dependent without clearly distinct size classes, integral projection models (IPM’s) are suitable for the integration and studying of population dynamics. The continuous state variable in IPM’s can be size variables like plant height or biomass. Many of the previous studies on clonal species have used discrete stage matrix models, but there has been some work on IPMs that include clonality [19–21]. As IPM’s allow researchers to study in more detail which plant size ranges contribute to e.g., the population growth of endangered or invasive species, IPM’s are also of use for applied ecology [22]. For instance, Hegland et al. constructed an IPM for the clonal shrub *Vaccinium* *myrtillus* to study the effect of ungulate grazing on population dynamics through various vital rates. Bruno et al. [20] studied interactions of gorgonian coral, a colonial, long-lived and modular animal, with a fungal pathogen. They examined the negative impact of the fungus and recovery of the coral and represents a framework to consider applying IPM’s to the interactions of biological control agents and Japanese knotweed. Here we collect demographic data, build, and analyze IPM’s with contrasting state variables, and discuss the usefulness of these and even more complex population models for underpinning management decisions.

## Materials and Methods

### Study system

**Figure 1:**
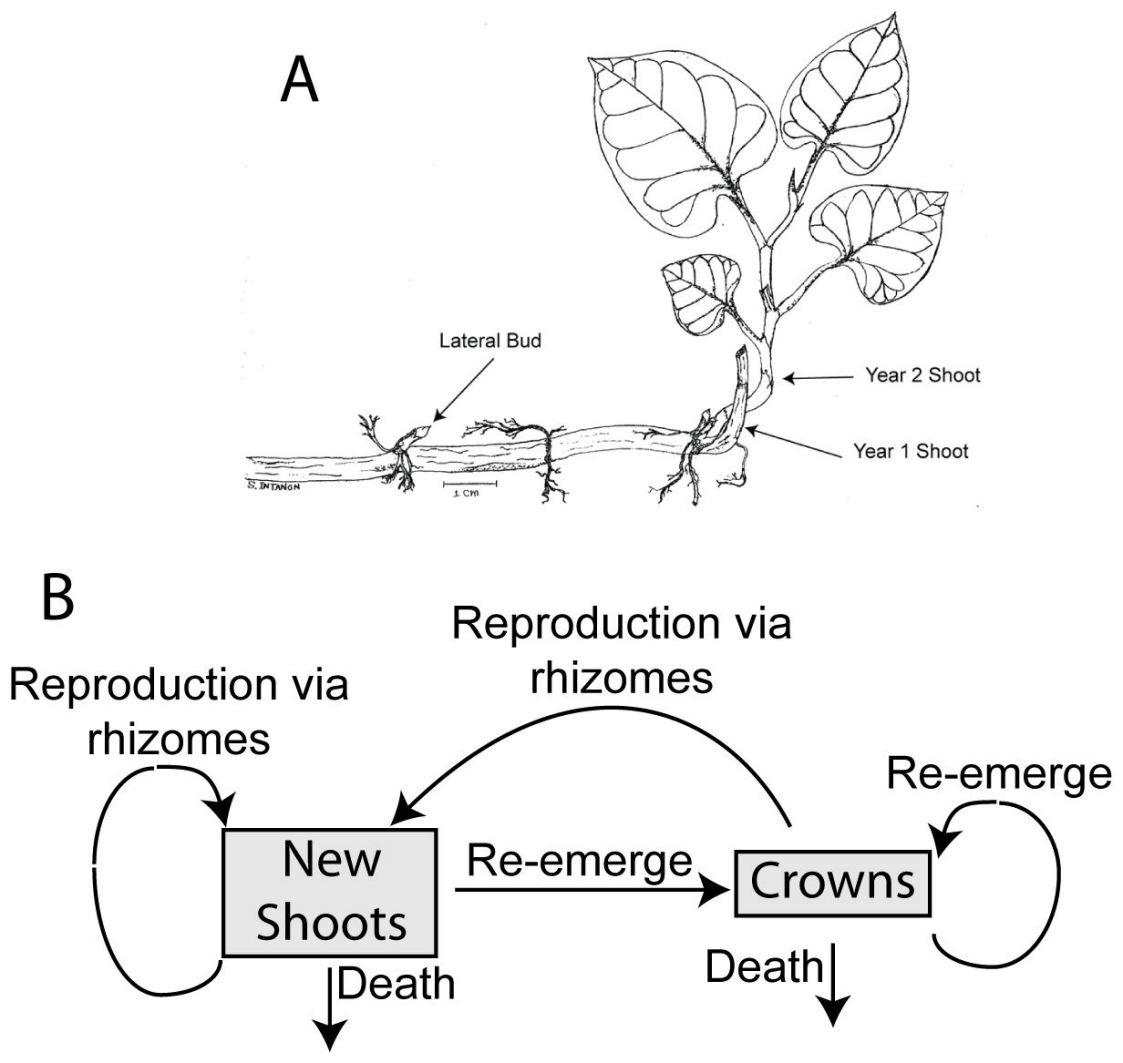
Japanese knotweed structures and life cycle. Japanese knotweed line drawing (A) showing above- and belowground structures relevant to population spread. Visible on the rhizome is a lateral bud. Drawing created by Suphannika Intanon. The life cycle diagram (B) of Japanese knotweed, showing the 2 stages considered in the integral projection model and the processes affecting individuals in each stage. Arrows represent a one-year time step.

*Polygonum* *cuspidatum* ( *Fallopia* *japonica* *,* *Reynoutria* *japonica* ) is a long-lived, perennial species that often invades riparian and highly disturbed areas. Seed production is a hotly debated issue for this species connected to its propensity to hybridize with *Polygonum* *sachalinense* [2,23]. U.S. populations of Japanese knotweed have been show to produce viable seeds [24], but it was only rarely observed in our populations. Seed establishment can be very low [14,25], and asexual reproduction through rhizomes is the likely source of spread [23,26]. Shoots emerge in spring and quickly reach heights of 1-4 m, flowering in mid-summer and senescing in autumn. Shoots begin to produce rhizomes in mid-summer, growing them throughout the summer (personal observation). At rhizome nodes, lateral and terminal buds form during the summer, possibly suppressed by apical dominance in the summer [27], and can emerge the following spring as new shoots (Figure 1A). After leaf senescence in autumn, shoot tissue dies but resources are held in storage belowground. Crowns were defined as shoots that emerged in the spring from the same location as an existing shoot from the previous autumn. Crowns were distinguished from new shoots that emerged in the spring at location greater than 5 cm from the location of an existing shoot in the previous autumn.

The life cycle of Japanese knotweed was simplified to include 2 stages: new shoots and crowns (Figure 1B). In a 1-year time step, new shoots either survive to return to become crowns, or die. Surviving crowns remain crowns. New shoots and crowns can also produce new shoots through rhizomes. We recognize the critical importance of detailed data on rhizome, bud, and seed production, and discuss (see *Discussion*) how the model would change to incorporate such state variables and processes to improve our understanding of Japanese knotweed population dynamics.

### Data collection

Data were collected over 2 years at 4 sites in southern Michigan, USA (Bath, Gull Lake, Maybury, and Sleepy Hollow). Individuals in southern Michigan are predominantly *P* *. cuspidatum* , identified using distinguishing characteristics outlined in Barney et al. [2]. *P* *. sachilinense* has been observed in northern Michigan, but was not observed near any of our sites. The study sites varied in soil and light characteristics and number of shoots present at the outset of the experiment (Table 1). Sites were selected based on accessibility for the duration of the study and size of the Japanese knotweed population. Two sites (Maybury and Sleepy Hollow) were located in State Parks and permission for the research was granted by the Michigan Department of Natural Resources. One site (Bath) was located on road right-of-way with permission obtained from the Clinton County Road Commission. The remaining site (Gull Lake) was located on private land and permission was obtained prior to study initiation. At Maybury and Sleepy Hollow, plants were selected from the exterior of the main population to more closely approximate the density of plants at other sites and to mimic a new, growing population. Densities ranged from 0.6 shoots m^-2^ at Bath to 2.7 stems m^-2^ at Maybury, which is similar to the density observed by Smith et al. [13] in their UK population.

**Table 1:** Site Characteristics.

**Site Name**	**Soil Texture**	**PAR (μmol m^-2^ s^-1^**)	**Area (m^2^**)	**Crowns Present at Outset**	**New Shoots Year 1**	**New Shoots Year 2**	**Total Plants**	**Rhizomes**	**Buds**
**Bath**	Sandy Clay Loam	131	20.8	12	41	51	104	63	182
**Gull Lake**	Sandy Loam	479	11.9	26	43	29	98	34	17
**Maybury**	Clay Loam	169	14.5	39	58	28	125	46	17
**Sleepy Hollow**	Sandy Loam	16	18.6	25	62	15	102	35	15

Site and ramet population data collected at 4 sites in southern Michigan, USA. Crowns emerged from the same location in consecutive years while new shoots emerged in a location where shoots had not been previously observed. Rhizome and bud counts were determined at the end of the 2-year study during excavation of each site. Photosynthetically active radiation (PAR) was measured at 1 m above ground level.

Generally, we followed the approach of Pitelka et al. [28], marking ramets each year and excavating the site after 2 years to identify connections among ramets and quantifying the age of rhizomes. Starting in April and continuing every 3 weeks until leaf senescence, all individual plants within the study site were marked, mapped, measured to the tallest leaf. Following leaf senescence, the stems were counted and biomass of each shoot was collected, dried at 70°C for 2 days, and weighed. Following leaf senescence in autumn of year 2, all sites were excavated and, to the extent possible, rhizomes were traced to any connected shoots. All rhizomes connected to other shoots (genets) were within 10 cm of the soil surface. Crowns rarely, if ever, form below 30 cm [13,29]. Although it is possible that plants were connected via rhizomes below 30 cm, we were unable to obtain permission for more extensive excavation.

### Data analysis

**Figure 2:**
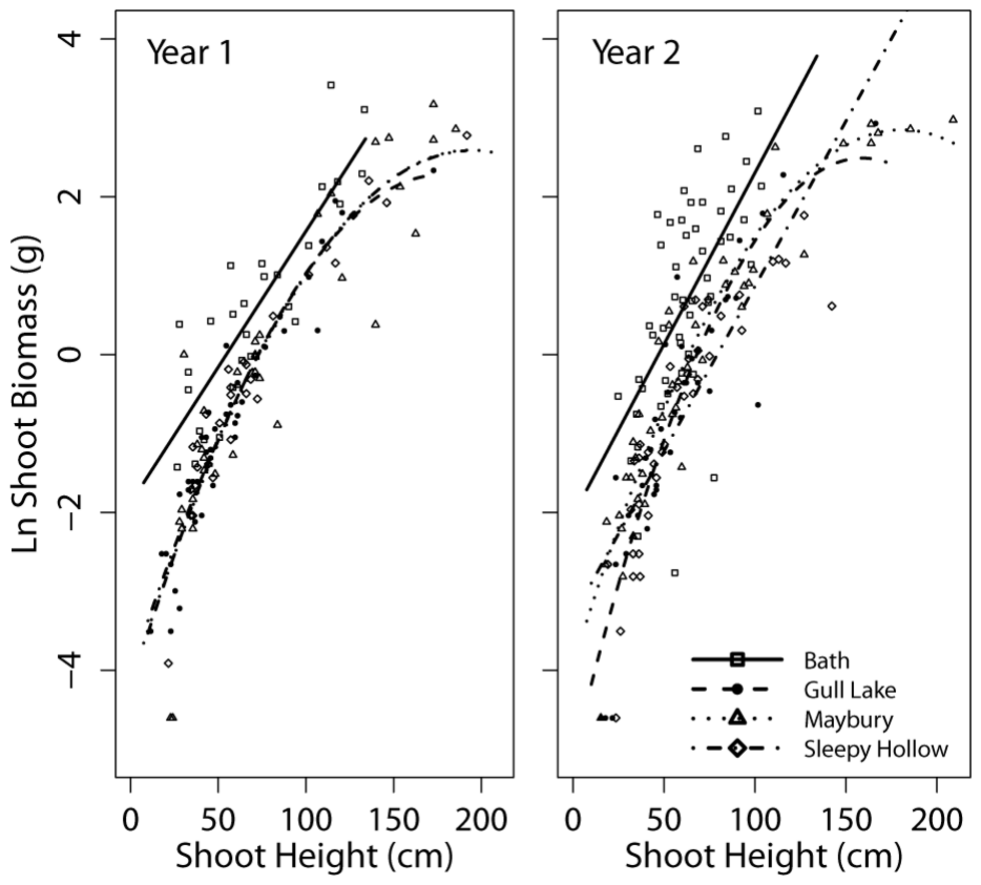
Shoot height and dry shoot biomass. Japanese knotweed biomass is strongly related to shoot height in both years of the study. Constant, linear, and quadratic models were fit to data for each site and the best fit was selected based on the lowest Akaike Information Criteria (AIC).

The main variables used in this model were shoot height to tallest leaf on September 15^th^ (before leaf senescence) and dry shoot biomass without any leaves. While these measures of shoot growth are independent, they are excellent predictors of one another (Figure 2). We were unable to use whole plant biomass because we waited until leaf senescence to ensure nutrients from the leaves had been reabsorbed by the crown which would have negatively affected year 2 growth. Constant, linear, and quadratic models were fit to data for each site and the best fit was selected based on the lowest Akaike Information Criteria (AIC). Analyses are presented for both shoot height and natural log (ln) of dry biomass to allow comparison and highlight instances when one or the other provides additional information. Data were separated by site because of the potential for major differences in population dynamics from variation in initial shoot density, soil conditions, and photosynthetically active radiation (PAR) (Table 1).

### IPM structure

An Integral Projection Model (IPM) is similar to transition matrix models and especially useful when the dynamics of populations are structured by continuous (rather than discrete) state variables [30,31]. IPMs were constructed from survival, growth, and fecundity (F) regression objects in R [using version 1.6 of The R package IPMpack; 32]. For each site we made 2 IPMs: one with shoot height as the continuous state variable, and one with ln shoot biomass as the continuous state variable.

The P object is the product of the survival and growth objects. Survival objects were generated by calculations based on survival of shoots from autumn of year 1 to autumn of year 2 and related to either state variable at the end of the growing season in year 1. Growth objects were constructed by relating each of the state variables (shoot height and ln shoot biomass) from year 2 to the same variable in year 1.

The fecundity object is a compilation of three vital rates: number of rhizomes produced, number of shoots per rhizome (New/Rhiz), and size of new shoots (New Size). We assumed any observed rhizome was produced during the summer of year 1 unless the rhizome was attached to a new year 2 shoot at which point the rhizome was assigned to that year 2 shoot. We are aware that rhizomes may have been produced earlier and there is a need to more definitively quantify timing of rhizome production (see *Discussion*). The New/Rhiz vital rate was defined as the number of new shoots in year 2 per known rhizome in year 1 and related to each of the state variables in year 1. Lastly, the New Size vital rate was defined as size of new year 2 shoots (only when connected to a year 1 shoot) and related to the state variables in year 1.

Relationships between state variables and vital rates were established by fitting constant, linear, and quadratic functions to the data, and selecting the best fit based on lowest Akaike Information Criteria (AIC). Growth and survival objects were multiplied together to give a P object (survival and growth) and added to a F object (fecundity) to construct an IPM for each site. Projected population growth rates (λ, i.e., the dominant eigenvalue) were calculated for each IPM.

### Elasticity and LTRE analyses

After constructing an IPM for each site, elasticity analysis provided details on the size range (shoot height and ln shoot biomass) having the greatest effect on λ. In stage-based matrix models elasticity identifies the transitions that are most important to the long-term growth rate. In IPM, there are a near continuous range of sizes, resulting in highest elasticity values over a range of sizes rather than of a specific size class. Similarly, when calculating the contribution of the P and F objects to λ, the contributions are represented as distributions over a range of sizes. A pseudo - life-table response experiment (LTRE) was used to identify the level of importance of the differences in 5 vital rates (growth, survival, number of rhizomes, new shoots/rhizome, new shoot size) for the differences in λ. To assess the median contribution to the λ-differences, we looked at each of the pair-wise comparisons between the 4 populations. For each pair we exchanged one of the vital rates between the 2 populations, and noted the resulting changes in λ. Therefore, 12 comparisons were made for each vital rate (each site (4) x every other site (3)) and the absolute value of the sum of the changes in λ was calculated to determine the relative contribution of each vital rate to the difference in λ between sites.

## Results

### Survival, Growth, Fecundity and Population Growth

**Figure 3:**
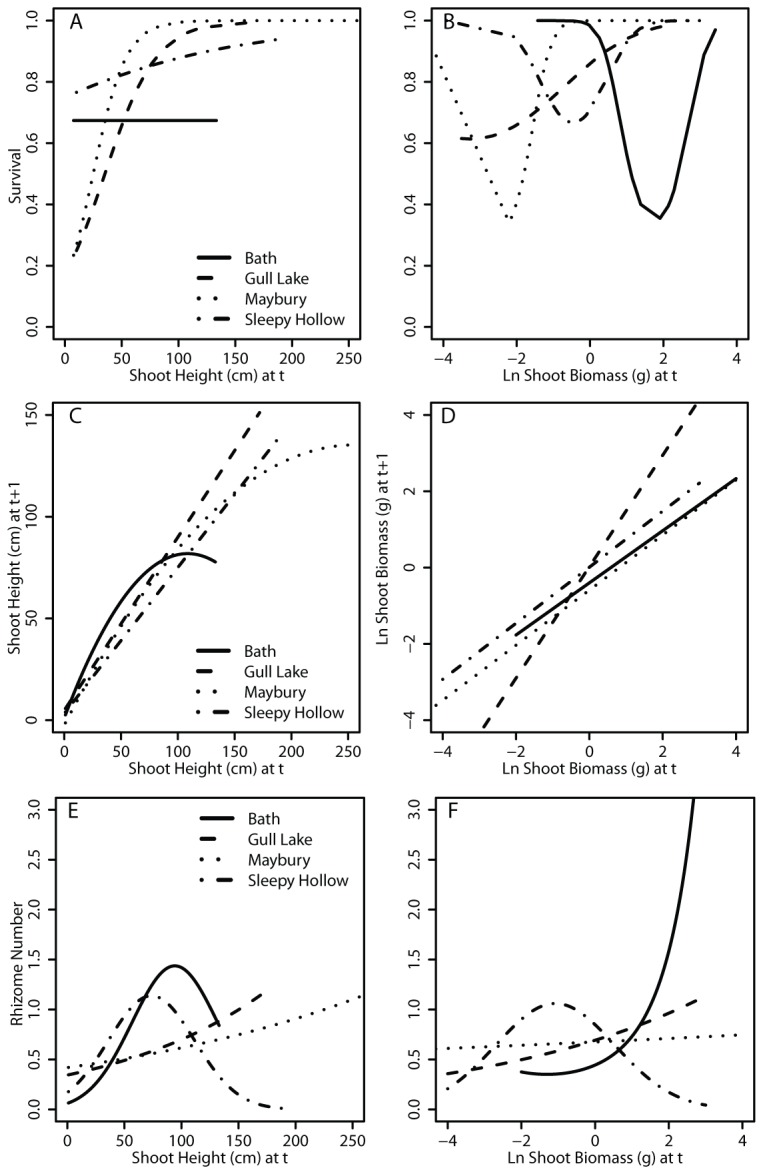
Survival, growth, and fecundity relationships. Shoot survival from year 1 to year 2 (A & B), shoot height at end of year 2 (C & D), and number of rhizomes connected to the shoot (E & F) as predicted by shoot height in year 1 and ln dry shoot biomass at end of year 1, respectively. Response curves are only shown for range of data at each site. Complete data representations are available in Appendix A.

Survival, growth, and number of rhizomes are dependent on the shoot height and shoot biomass in year 1 (Figure 3). Survival increased with increasing shoot height (Figure 3A) for all sites except Bath where survival was not height dependent (Figure S1 for site data). In contrast, when exploring survival as a function of ln shoot biomass, only Gull Lake had increasing survival as ln shoot biomass increased (Figure 3B). Bath, Maybury, and Sleepy Hollow showed lowest survival for intermediate biomass with high survival for low and high biomass shoots (Figure S1).

Growth from year 1 to year 2 was generally linear across all sites, whether using shoot height or ln shoot biomass (Figure 3C and 3D). Bath and Maybury show a quadratic response to shoot height with shoots slightly less than the maximum observed at each site showing the greatest size increases to year 2. Gull Lake showed the greatest year 1 to year 2 increase in shoot biomass (Figure 3D).

Number of rhizomes varied widely among sites (Figure 3E and 3F). While Gull Lake and Maybury showed exponentially increasing number of rhizomes with greater shoot height, Bath and Sleepy Hollow had greatest fecundity by intermediate height plants (Figure 3E). Bath and Gull Lake had exponentially increasing fecundity as shoot biomass increased while Maybury had only linear increase as a function of shoot biomass (Figure 3F). Intermediate sized individuals at Sleepy Hollow produced the greatest number of rhizomes.

Population growth rates projected by the IPM’s showed a range from populations declining slightly to others growing rapidly (Table 2). Numbers of shoots were projected to increase by 13-233% from year to year. Population growth rates based on shoot height were 12-25% less than population growth rates based on shoot biomass.

**Table 2:** Projected Population Growth Rates (λ) for 4 sites based on shoot height and dry shoot biomass at the end of year 1.

	Bath	Gull	Maybury	Sleepy Hollow
Shoot Height	2.50	0.92	1.06	1.08
Ln (Shoot Biomass)	3.33	1.13	1.20	1.25

Rates greater than 1 project population growth.

### Elasticity Analysis

**Figure 4:**
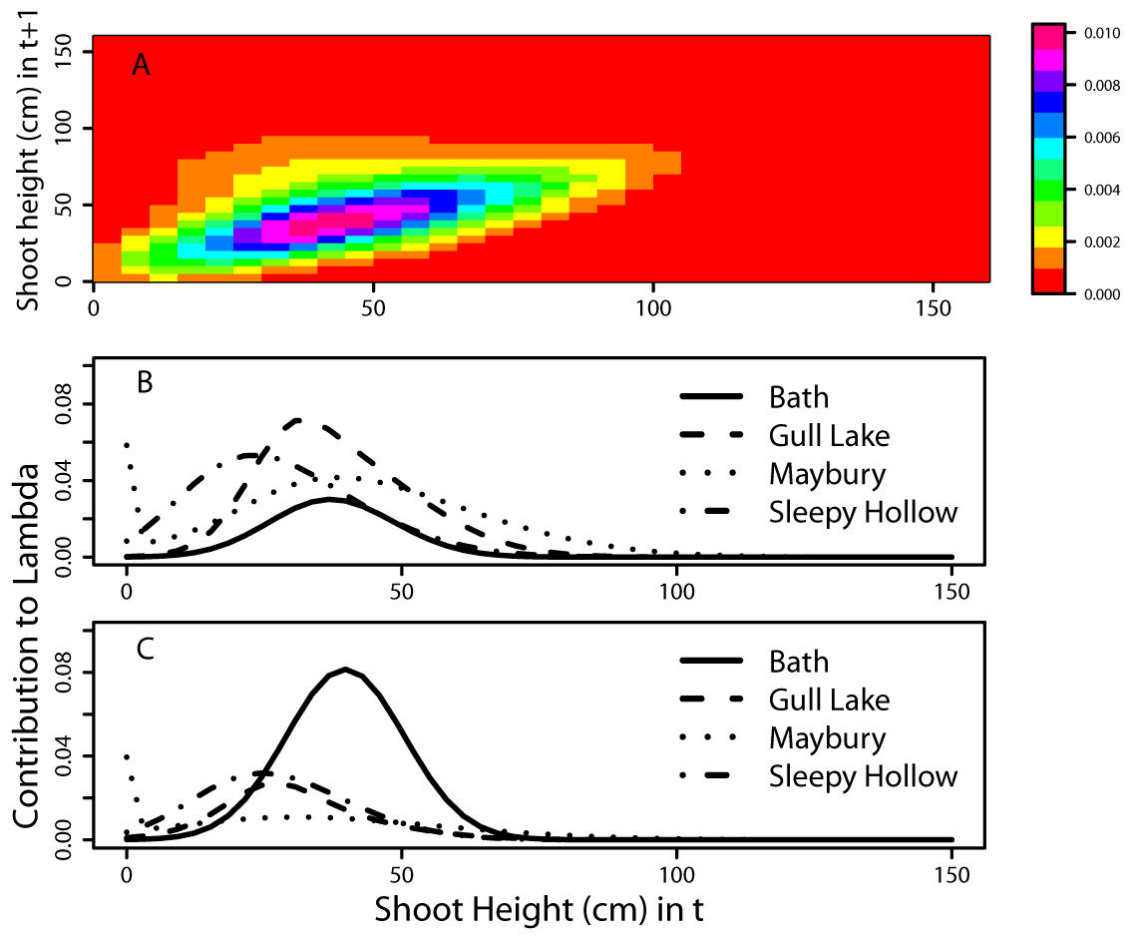
Elasticity and Contributions. Elasticity (A) of IPM matrix for Sleepy Hollow show shoots with low to medium heights (approximately 30-80 cm) have the greatest impact on the population growth rate. Sleepy Hollow is used as an example, see Appendix B for elasticity analysis for other sites. Generally, there was a greater contribution of the P object (B, survival and growth) than the F object (C, fecundity) to the population growth rate, as determined by the area under the curve. The exception is the Bath site where the F object had a greater contribution.

Elasticity represents the relative contributions of different transitions, which can be summarized per stage class. With continuously varying stage classes, elasticity values identify the relative contribution of a range of shoot sizes to population growth rates (Figure 4A). For example, the population growth rate at Sleepy Hollow was dependent on the small to medium sized (~30 to 80 cm) ramets. The same was generally true for the three other populations; medium sized individuals lying roughly on the 1:1 growth line had the greatest elasticity values (Figure S2 for site data).

Elasticity can be decomposed to consider the contribution of the P object (survival and growth objects) and the F (fecundity) object. Survival and growth had a greater contribution than fecundity to population growth rates at Gull Lake, Maybury, and Sleepy Hollow (Figure 4B and 4C). At the Bath site, fecundity overwhelmingly affected the high population growth rate, which is expected for populations which are increasing in size [33] (Table 2, Figures 4B & 4C).

### LTRE Analysis

**Figure 5:**
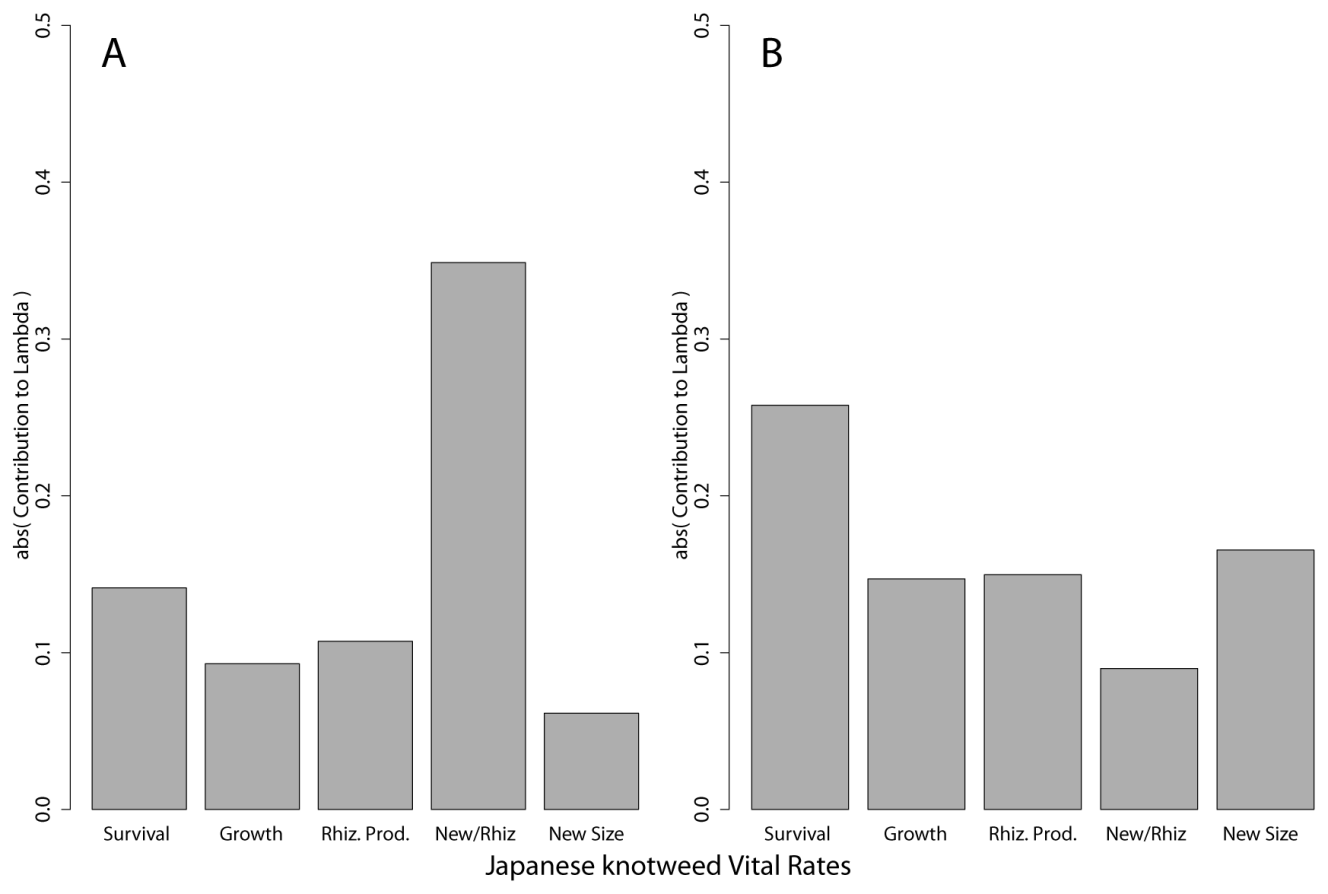
Contributions of Vital Rate Changes. Median absolute value of potential contribution a change in a vital rate could have on population growth rates (λ) across all sites based on final plant height (A) and dry shoot biomass (B). Contributions were calculated using each site as a reference location for every other site, and varying a single vital rate at a time. Fecundity is separated into rhizome production (Rhiz. Prod.), number of new shoots in year 2 per rhizome (New/Rhiz), and the shoot height of new, connected shoots (New Size). See Appendix C for complete data.

We conducted an LTRE-like analysis to determine the contribution of vital rates to the differences in population growth rates among sites (Figure 5) for each state variable in year 1. When using shoot height as the state variable, the ratio of new shoots to the number of rhizomes overwhelmingly affected to projected population growth rates. The remaining vital rates – survival from year 1 to year 2, number of new rhizomes in year 2 per extant shoot in year 1, and final shoot height of new shoots connected to extant shoots in year 1, all contributed far less. Growth, i.e., change in shoot height from year 1 to year 2, had little effect on the variation in population growth rate. When using shoot biomass as the state variable, no single vital rate affected the projected population growth rates although survival from year 1 to year 2 was greater than the others. In contrast to shoot height, the number of new shoots per rhizome had the lowest effect. There was an order of magnitude difference in effect when comparing sites to Bath (Table S1 for site comparisons), most likely due to variation in number, size, and connectedness of rhizomes at Bath compared to other locations.

## Discussion

Japanese knotweed population dynamics varied among the 4 sites when considering projected population growth rates, λ-elasticity, and the impact of vital rate differences on variation in λ. No two sites had similar growth, survival, or fecundity relationships, although Gull Lake and Maybury are more similar than the others (Figure 3). Some sites showed medium-sized plants as having a greater impact on population growth than small or large plants, which differs from other clonal plants that are more fecund and have greater survival with increasing age or size [34,35]. Interestingly, Suzuki [11] found growth patterns of large populations (area > 31 m^2^) had greater variability in growth rates than small populations. It is possible our populations were just at a different stage of development (some early, others late); where collecting data from more sites could yield a greater range of population growth rates.

There were also differences in PAR and soil texture that may have impacted plant growth (Table 1). Sleepy Hollow had the lowest PAR among sites. However, plants at Sleepy Hollow did not have a significantly lower biomass and had a similar number of rhizomes per plant compared to sites with much higher PAR suggesting Japanese knotweed plants are able to grow irrespective of PAR. Potentially the foliage structure was different among sites as found by Suzuki [11]. However, the densities recorded by Suzuki were 2 orders of magnitude greater than our densities and it is unlikely plants in our study were affected by intraspecific light competition to change their foliage structure, although we did not measure structure in order to verify. Bath did have soil and disturbance conditions that differed from the other 3 sites. The Bath population was located on the edge of a heavily traveled gravel road. In 2011 and 2012, the road was graded once in July to smoothen the roadbed, potentially creating and moving rhizome fragments from a larger population ‘upstream’ of the study site. No new shoots emerged after the disturbance, but it may have resulted in more new shoots in the following year and potentially activating new rhizome production similar to cutting [36]. Jongejans et al. [15] and potentially our study found disturbance facilitates clonal reproduction (see Bath site). They advocate increasing disturbance to increase the growth of the endangered *Cirsium* *dissectum* , while we advocate consideration for how reduced disturbance may impact growth of an invasive species.

Temperature fluctuations also may have impacted knotweed growth and survival. Average emergence date of shoots was 14 days earlier in 2012 compared to 2011 due to an early season warming trend. This was followed by a series of hard frosts in April and May 2012 that affected sites differently. At more densely forested sites (Maybury and Sleepy Hollow), 37% (n=104) and 46% (n=87) of shoots were damaged compared to 11% (n=87) and a single shoot at Bath and Gull Lake, respectively. Crowns may keep lateral buds from producing new rhizomes or shoots [12] and the loss of apical dominance, even for a short period in the spring, may have allowed additional shoots to emerge. Nearly 70% of damaged shoots were able to regrow a new shoot, but the loss of nutrients to a failed shoot may have set back the surviving plants just when growth rate is the greatest [11]. Overall, there were fewer new shoots in 2012 compared to 2011 at Maybury and Sleepy Hollow, probably because any additional shoots released from apical dominance were quickly killed by the series of frosts. This left crowns with sufficient nutrient reserves to produce a 2^nd^ or 3^rd^ shoot and a few of the most resilient new shoots.

Japanese knotweed genet growth has been simulated for individual populations [12,13] but not connected to its life cycle to generalize for many populations. There have been efforts to combine demographic and population models for perennial plants using both stage-structured matrix models and IPM’s. The limitation of stage-structured matrix models was highlighted by Lyngstad et al. [16] when they suggested classifying by size may have influenced their results. In contrast, IPMs use continuous state variables and may be a better fit for some perennial plants that may not have specific stage classes. IPM’s have been used to identify growth and survival of ramets [19] or a balance of sexual and asexual reproduction [21] as a greater contributor to population growth rate. For some perennial species, the tradeoff between sexual and asexual reproduction can have a dramatic effect on growth rates, where some seed production may be essential to population stasis or growth [16,21]. A recent study found Japanese knotweed populations in the Northeastern US use both sexual and asexual reproduction and had a surprising amount of genetic diversity [23]. This was not the case in our study where seeds were infrequently produced and had low viability. There is a distinction between the quantity of offspring possible with sexual reproduction and the quality of offspring possible with asexual reproduction and the possibility to affect population dynamics. The contribution of seed to our populations was negligible. But the underlying conceptual model (Figure 1) could be modified to accommodate populations where sexual reproduction is more prevalent [24]. The possibility remains for Japanese knotweed populations to rely on sexual reproduction as a critical means of population growth [23] and must be explored in locations where both sexual and asexual reproduction occurs.

**Figure 6:**
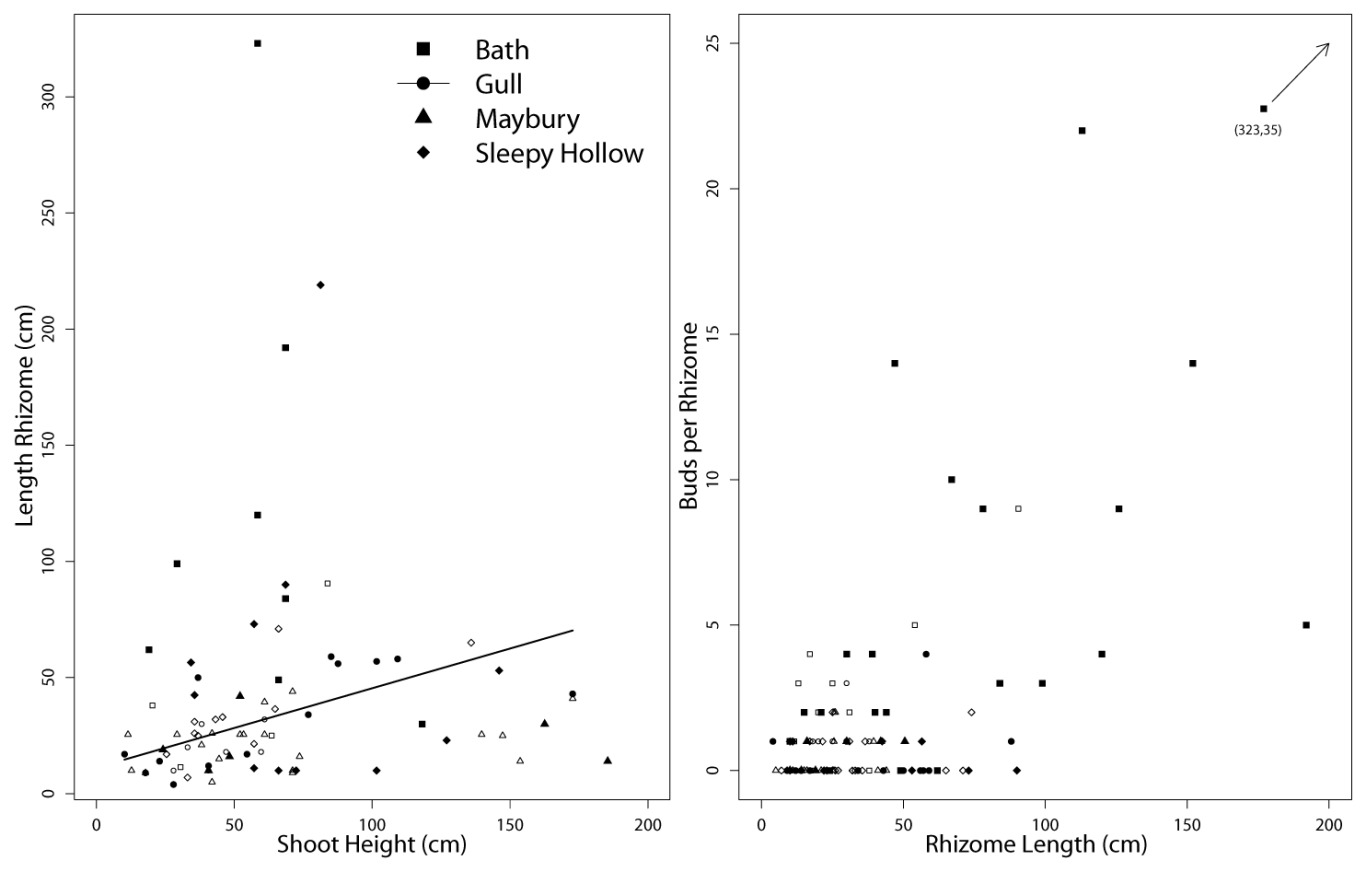
Rhizome and Bud Production. The number of visible lateral and terminal buds present on excavated rhizomes (solid symbols). Snapped rhizomes’ lengths (open symbols) are approximate because they could not be followed to their terminal bud.

In an ideal world, we would have been able to more completely describe the life cycle of Japanese knotweed and increase the model complexity. In our current model, the entire rhizome is treated as a reproductive output because of the small amount of rhizome tissue needed for a new shoot to form [6]. We also considered using rhizome length (instead of rhizome number) as predicted by shoot height (Figure 6A). One site (Gull Lake) had a significant relationship between shoot height and rhizome length, but all sites had considerable variation in rhizome length. This was partly due to the incomplete measurement of many rhizomes. Between 29% (Bath, n=35) and 66% (Maybury, n=35) of rhizomes were incompletely measured because of rhizomes grew deeper than 25 cm or researcher error (inadvertently cutting the rhizome). We called these ‘snapped’ rhizomes and they were shorter (27 cm, SE=2.3) than fully measured rhizomes (57 cm, SE=6.4). Rhizome decay may also have contributed to the reduced number of observed rhizome connections [37,38]. A more complete excavation [possibly using compressed air a la 13] may reveal greater number of connections and the true length of rhizomes. Given a more complete data set of rhizome lengths and when rhizomes were produced and died, rhizome length may have been used instead of number of rhizomes to better quantify the fecundity object (F) and the asexual contributions to population growth. Our population model could then be combined with previous spatial models of Japanese knotweed growth [13] to more accurately predict spatial and temporal population dynamics.

Despite the challenge associated with rhizome excavation, we advocate for greater attention to the demography of buds as an estimate of belowground fecundity, since they will become new rhizomes and shoots. We observed lateral and terminal buds in various stages of apparent maturation. Some buds were enlarged and resembled buds present on the crowns in the autumn, suggesting shoot production in the following year (Figure 1 for visual reference) while other resembled swollen nodes with unknown emergence dates. We counted the number of buds, regardless of type, on each rhizome and related it to rhizome length (Figure 6B). Firstly, like the rhizome length, the true count of bud production was limited by our ability to excavate the rhizome network and therefore prevented an estimation of buds m^-2^, a measure of potential growth. Secondly, the inclusion of buds into the model was restricted by lack of knowledge about bud demography. We were able to estimate time of bud production, but had no data on bud death, dormancy, or likelihood of producing a new shoot. Reportedly, buds remain dormant as long as the crown is alive [39], however, this would preclude new shoots connected to crowns which we observed at all sites, albeit in lower numbers than previously reported [11,13]. Since buds specifically give rise to new shoots (similar to seeds), they provide a crucial component in a more complete life cycle analysis for this species.

Lastly, inclusion of spatial and temporal variation in growth may improve the generalizability of the model. For example, combining these IPM’s with spatially explicit ramet growth models [12,13] would significantly improve each one and expand our knowledge of spatial patterns of population growth. Our current knowledge of Japanese knotweed demography is limited to less than 10 sites with different data on growth, survival, and fecundity [6,13,14,29]. There continues to be a need to more widely measure and model Japanese knotweed demography to make generalizations about vital rates. We tried to parameterize periodic IPM’s to better capture the temporal division of resource allocation – aboveground growth in spring, belowground growth in summer and autumn, but were not able to completely parameterize both matrices. Timing and rate of rhizome growth are rarely reported and often estimated based on total rhizome length and approximate date of initiation. To the best of our knowledge, these data do not exist for Japanese knotweed and are likely site specific. Repeated excavation can quantify timing of belowground growth and support periodic IPM’s that can better predict intra-annual Japanese knotweed population dynamics.

Building multi-site plant population models like the one presented is a first step towards using these models to inform management. In particular, plant models help identify the critical transitions for the plant population to grow. Japanese knotweed populations in our study relied on asexual production of new shoots per rhizome (Fig. 5) to grow in population size. Because this transition occurred less frequently at Sleepy Hollow, Maybury, and Gull Lake, those populations grew more slowly. Clearly the population at Bath, where shoots per rhizome were the highest, had a concomitantly higher population growth rate. Therefore, managers should consider this a vulnerable life cycle transition and potential management target. Unfortunately, chemical control for perennial species, and Japanese knotweed in particular, take place in autumn because the rhizome is a principal carbon sink during this time [40]. This is unfortunate because the rhizome has already been produced starting in June or July (unpublished data). Depending on the length of the rhizome and strength of the buds as carbon sinks at timing of treatment, herbicides may be more or less effective at targeting the number of new shoots per rhizome. In a related study, we found 0, 2, and 5 buds on rhizomes of 3 shoots treated with glyphosate compared to 0 buds on rhizomes of shoots treated with imazapyr and imazamox, systemic herbicides labeled for Japanese knotweed management in the US. Imazapyr may be effective at reducing carbon transport to the buds in autumn [39], and imazamoz may act similarly. If these chemicals are targeting buds as sinks, then the next step in closing the gap between data and modeling is determining the effect of herbicides on the timing and number of buds produced for a rhizome and the impact on the life cycle of Japanese knotweed. Similarly, if biological control agents are to be effective on Japanese knotweed [7,8], we must investigate which life cycle transitions the agent affects and the implications for population growth. Managers will then be able to decide, based on data and models, about chemical and biological control of Japanese knotweed.

## Supporting Information

Figure S1 **Growth, Survival, and Fecundity Objects used to build site-specific integral projection models.**
(DOCX)

Figure S2 **Elasticity of Japanese knotweed integral projection models.**
(DOCX)

Table S1 **Life-Table Response Experiment (LTRE) results.**
(DOCX)
