# Supplementary material for: Elucidating the Population Dynamics of Japanese Knotweed Using Integral Projection Models
Source: PLoS One. 2013 Sep 20;8(9):e75181. doi: 10.1371/journal.pone.0075181 (PMC3779190; doi:10.1371/journal.pone.0075181)
Supplement: Figure S1 — Growth, Survival, and Fecundity Objects used to build site-specific integral projection models. (DOCX) [file pone.0075181.s001.docx]

**Elucidating the population dynamics of Japanese knotweed using integral projection models**

Joseph Dauer and Eelke Jongejans

Figure S1. Survival, Growth, and Fecundity Objects used to build site-specific integral projection models.

This appendix contains figures that display the data to which survival, growth and fecundity objects are fitted, using the R package *IPMpack* (Metcalf et al. 2013). For each of these three vital rates we show eight panels: for the four sites (Bath, Gull Lake, Maybury and Sleepy Hollow), times the two continuous state variables (plant height and biomass). The fitted objects contain (generalized) linear regression models, which are represented by the lines in the panels, as well as by the regression function given underneath the panels.

Literature:

Metcalf CJE, McMahon SM, Salguero-Gómez R, Jongejans E (2013) IPMpack: an R package for integral projection models. Methods in Ecology and Evolution 4: 195–200.

S1.1 Survival Objects by final Plant Height

Bath – ln(surv/1-surv) = 0.7, Null Dev – 58.1

Gull Lake – ln(surv/1-surv) = -1.5 + 0.04*ht_t_, Null Dev – 71.1

Maybury – ln(surv/1-surv) = -1.7 + 0.07*ht_t_, Null Dev – 87.9

Sleepy Hollow – ln(surv/1-surv) = 1.1 + 0.009*ht_t_, Null Dev – 37.5

S1.2 Survival Objects by ln Biomass

Bath – ln(surv/1-surv) = 4.1 – 5.3*bm_t_ + 1.5*bm_t_ ^2^, Null Dev – 29.1

Gull Lake – ln(surv/1-surv) = 1.8 – 0.8*bm_t_ + 0.1*bm_t_ ^2^, Null Dev – 46.6

Maybury – ln(surv/1-surv) = 9.0 – 7.7*bm_t_ + 1.5*bm_t_ ^2^, Null Dev – 29.6

Sleepy Hollow – ln(surv/1-surv) = 0.9 – 0.8*bm_t_ + 0.9*bm_t_ ^2^, Null Dev – 26.7

S1.3 Growth Objects by final Plant Height

Bath – ht_t+1_=1.1+1.5*ht_t_-0.01ht_t_^2^, sd=18.2

Gull Lake - ht_t+1_=4.9+0.9*ht_t_, sd=15.9

Maybury - ht_t+1_=-2.4+1.1*ht_t_-0.002ht_t_^2^, sd=34.7

Sleepy Hollow - ht_t+1_=3.3+0.7*ht_t_, sd=13.0

S1.4 Growth Objects by ln Biomass

Bath – ht_t+1_ = 0.5 + 0.5*ln(bm_t_), sd=1.3

Gull Lake - ht_t+1_ = -0.1 + 1.1*ln(bm_t_), sd=1.2

Maybury - ht_t+1_ = -0.6 + 7*ln(bm_t_), sd=1.1

Sleepy Hollow - ht_t+1_ = -0.7 + 0.7*ln(bm_t_), sd=0.7

S1.5 Fecundity Objects by final Plant Height

Bath – log(rhiz_t_)= -2.8+0.07*ht_t_ -3E-4*ht_t_^2^, Null Dev - 127

Gull Lake - log(rhiz_t_) = -1.1+0.007*ht_t_, Null Dev – 41

Maybury - log(rhiz_t_)= -0.9+0.004*ht_t_, Null Dev - 67

Sleepy Hollow - log(rhiz_t_)= -1.8+0.05*ht_t_ -4E-4*ht_t_^2^, Null Dev - 49

S1.6 Fecundity Objects by ln Biomass

Bath – log(rhiz_t_)= -0.8+0.4*lm(bm_t_) +0.1* lm(bm_t_)^2^, Null Dev - 104

Gull Lake - log(rhiz_t_) = -0.4+0.1* lm(bm_t_), Null Dev – 40

Maybury - log(rhiz_t_)= -0.4+0.03* lm(bm_t_), Null Dev - 48

Sleepy Hollow - log(rhiz_t_)= -0.005 - 0.2* lm(bm_t_) – 0.1*ht_t_^2^, Null Dev - 32
