# Supplementary material for: Elucidating the Population Dynamics of Japanese Knotweed Using Integral Projection Models
Source: PLoS One. 2013 Sep 20;8(9):e75181. doi: 10.1371/journal.pone.0075181 (PMC3779190; doi:10.1371/journal.pone.0075181)
Supplement: Figure S2 — Elasticity of Japanese knotweed integral projection models. (DOCX) [file pone.0075181.s002.docx]

**Elucidating the population dynamics of Japanese knotweed using integral projection models**

Joseph Dauer and Eelke Jongejans

Figure S2. Elasticity of Japanese knotweed integral projection models.

This appendix contains λ-elasticity plots of the constructed IPM’s, showing how much the transitions in a large matrix-representation of the IPM’s contribute to projected population growth (λ). These elasticity plots are shown per site – state variable (plant height or ln biomass). The coloring is standardize between sites for each of the two state variables separately. The elasticity matrices were constructed with the R package *IPMpack* (Metcalf et al. 2013) and depicted with the *image.plot* function of the *fields* package (Fields Development Team 2006).

Literature:

Fields Development Team (2006). fields: Tools for Spatial Data. National Center for Atmospheric Research, Boulder, CO. www.cgd.ucar.edu/Software/Fields.

Metcalf CJE, McMahon SM, Salguero-Gómez R, Jongejans E (2013) IPMpack: an R package for integral projection models. Methods in Ecology and Evolution 4: 195–200.

S2.1 Elasticity by final Plant Height

S2.2 Elasticity by ln Biomass
