# Supplementary material for: Elucidating the Population Dynamics of Japanese Knotweed Using Integral Projection Models
Source: PLoS One. 2013 Sep 20;8(9):e75181. doi: 10.1371/journal.pone.0075181 (PMC3779190; doi:10.1371/journal.pone.0075181)
Supplement: Table S1 — Life-Table Response Experiment (LTRE) results. (DOCX) [file pone.0075181.s003.docx]

**Elucidating the population dynamics of Japanese knotweed using integral projection models**

Joseph Dauer and Eelke Jongejans

Table S3. Life-Table Response Experiment (LTRE) results.

This appendix shows the details of the life table response experiments that we performed. For each of the two state variables (plant height and ln biomass) we made all possible pair-wise site comparisons. Site 1 is the reference site and Site 2 is comparison site.

S3.1 LTRE by final Plant Height

S3.2 LTRE by natural log biomass
